# TOWARD A NEUROECOLOGICAL MODEL OF FINANCIAL CAPACITY AMONG OLDER ADULTS

**DOI:** 10.1093/geroni/igac059.1752

**Published:** 2022-12-20

**Authors:** Michael Barnett, Samuel Van Vleet, Kailee Prentice, Annika Wurm, Danica Bass, Yenifer Morales Mejia, Jeanné Dube, Samuel Van Vleet

**Affiliations:** The University of Texas at Tyler, Tyler, Texas, United States; Miami University, Oxford, Ohio, United States; The University of Texas at Tyler, Tyler, Texas, United States; The University of Texas at Tyler, Tyler, Texas, United States; The University of Texas at Tyler, Tyler, Texas, United States; The University of Texas at Tyler, Tyler, Texas, United States; The University of Texas at Tyler, Tyler, Texas, United States; Miami University, Oxford, Ohio, United States

## Abstract

In recent decades, technology has changed how individuals interact with their money and with each other. A combination of financial vulnerability and low technological literacy puts many older adults at risk for identity theft, fraud, and financial exploitation. We reviewed the literature on financial capacity, financial exploitation, and digital literacy. Extant models and measures of financial capacity among older adults emphasize numeracy and basic functional skills, such as writing checks and counting change; these may not reflect the digital nature of contemporary financial activity. We propose the neuroecological model of financial capacity among older adults. This function-led model contends that financial capacity consists of neurocognitive abilities to make sound financial decisions in a complex environment, to use technology to monitor and carry out financial activities, and the ability to protect personal information and guard against fraud. This points to a need for more ecologically valid measures of financial capacity and vulnerability to financial exploitation that addresses the role of technology in everyday financial activities.

